# Supplementary material for: Walking Speed is the Sole Determinant Criterion of Sarcopenia of Mild Cognitive Impairment in Japanese Elderly Patients with Type 2 Diabetes Mellitus
Source: J Clin Med. 2020 Jul 6;9(7):2133. doi: 10.3390/jcm9072133 (PMC7408848; doi:10.3390/jcm9072133)
Supplement: Supplementary file 1 [file jcm-09-02133-s001.zip › S2.pdf]

Supplement 2. Baseline characteristics of subgroups of studied patients with type 2 diabetes mellitus

	Age < 60 years n = 109	Age ≥ 60 years n = 329	*P*	Men n = 244	Women n = 194	*P*	BMI < 25 n = 172	BMI ≥ 25 n = 266	*P*
Age, years	51 [45, 55]	71 [67, 76]	<0.01	69 [62, 75]	66 [58, 73]	<0.05	71 [66, 77]	65 [54, 71]	<0.01
Male, n (%)	78 (48)	166 (60)	<0.05				106 (62)	138 (52)	<0.05
MOCA-J, points	27 [26, 29]	25 [22, 27]	<0.01	26 [23, 28]	26 [23, 27]	0.82	25 [23, 27]	26 [23, 28]	0.07
MOCA-J < 26 points, n (%)	24 (22)	193 (58)	<0.01	122 (50)	95 (49)	0.67	94 (55)	123 (46)	<0.01
**Anthropometry**									
Systolic blood pressure, mmHg	128 [116, 138]	134 [122, 145]	<0.01	131 [119, 142]	134 [122, 146]	<0.05	129 [120, 143]	134 [121, 144]	0.17
Diastolic blood pressure, mmHg	77 ± 11	71 ± 11	<0.01	74 ± 12	73 ± 12	0.15	72 ± 11	75 ± 12	<0.01
Body weight, kg	79.8 [67.5, 92.3]	61.2 [54.5, 71.9]	<0.01	67.3 [59.0, 79.5]	61.9 [51.0, 74.8]	<0.01	56.0 [49.5, 60.0]	74.5 [65.9, 84.9]	<0.01
BMI, kg/m^2	29.8 [26.7, 34.7]	24.5 [21.8, 27.7]	<0.01	24.7 [22.0, 28.1]	26.6 [22.7, 31.2]	<0.01	21.7 [20.4, 23.0]	28.3 [25.8, 32.1]	<0.01
Waist circumference, cm	100.5 [89.9, 111.8]	88.0 [80.3, 97.0]	<0.01	89.9 [82.0, 99.5]	91.2 [82.0, 102.1]	0.3	80.1 [76.3, 85.0]	98.0 [91.1, 105.1]	<0.01
Fat mass, kg	29.7 [21.8, 39.9]	18.6 [13.6, 25.5]	<0.01	18.9 [13.3, 25.6]	23.3 [16.1, 32.4]	<0.01	13.4 [10.5, 15.6]	27.1 [21.5, 33.8]	<0.01
Fat ratio, %	37.6 [29.0, 45.5]	31.0 [24.0, 37.1]	<0.01	27.9 [22.1, 34.1]	38.1 [32.2, 45.4]	<0.01	23.5 [18.8, 29.3]	37.2 [31.8, 43.7]	<0.01
Hand grip strength, kg	37.0 [28.5, 47.5]	30.0 [23.0, 40.0]	<0.01	40.0 [33.0, 46.0]	25.0 [20.0, 29.0]	<0.01	31.0 [23.3, 40.0]	33.0 [25.0, 42.0]	<0.05
Walking speed, m/sec	1.67 [1.43, 1.82]	1.54 [1.33, 1.67]	<0.01	1.67 [1.43, 1.82]	1.54 [1.33, 1.67]	<0.01	1.54 [1.36, 1.82]	1.54 [1.43, 1.82]	0.73
Muscle mass, kg	48.8 [41.2, 52.9]	41.5 [34.9, 46.7]	<0.01	47.1 [42.9, 51.0]	35.9 [32.5, 40.2]	<0.01	39.3 [34.2, 44.7]	45.0 [37.8, 50.6]	<0.01
Skeletal muscle index, kg/m^2	7.8 [7.0, 8.7]	6.9 [6.2, 7.6]	<0.01	7.6 [7.0, 8.3]	6.4 [5.8, 7.1]	<0.01	6.6 [5.9, 7.2]	7.6 [6.8, 8.3]	<0.01
Sarcopenia, n (%)	2 (1)	36 (13)	<0.01	16 (7)	22 (11)	<0.05	19 (11)	19 (7)	<0.05
**Comorbidities**									
Duration of diabetes, years	11 [6, 15]	16 [10, 22]	<0.01	15 [9, 22]	13 [8, 19]	<0.05	15 [8, 23]	14 [9, 20]	0.25
Hypertension, n (%)	105 (65)	199 (72)	0.11	169 (69)	139 (70)	0.94	106 (62)	198 (74)	<0.01
Dyslipidemia, n (%)	118 (73)	184 (67)	0.20	161 (66)	141 (73)	0.15	101 (59)	201 (76)	<0.01
Coronary heart diseases, n (%)	18 (11)	46 (17)	0.11	48 (20)	16 (8)	<0.01	23 (13)	41 (15)	0.56
Stroke, n (%)	14 (9)	22 (8)	0.81	23 (9)	13 (7)	0.3	14 (8)	22 (8)	0.96
**Life habits**									
Regular walking, n (%)	57 (35)	116 (56)	0.17	101 (41)	72 (37)	0.39	74 (43)	99 (37)	0.21
Current or ex-smoker, n (%)	93 (57)	138 (50)	0.89	183 (75)	48 (25)	<0.01	95 (55)	136 (51)	0.1
Current or ex-drinker, n (%)	61 (38)	134 (49)	<0.05	156 (64)	39 (20)	<0.01	86 (50)	109 (41)	<0.01
**Blood measurements**									
Albumin, g/dL	4.4 [4.1, 4.6]	4.2 [4.0, 4.4]	<0.01	4.3 [4.1, 4.5]	4.2 [4.0, 4.4]	0.18	4.3 [4.0, 4.5]	4.2 [4.0, 4.4]	0.65
AST, U/L	22 [17, 32]	21 [17, 27]	0.47	22 [18, 29]	20 [17, 26]	<0.05	20 [17, 26]	22 [17, 30]	0.05
ALT, U/L	24 [17, 46]	18 [13, 26]	<0.01	20 [14, 31]	18 [13, 27]	<0.05	17 [12, 22]	21 [15, 35]	<0.01
γGP, U/L	31 [19, 57]	23 [17, 35]	<0.01	27 [19, 45]	22 [15, 34]	<0.01	21 [15, 32]	27 [18, 44]	<0.01
Fasting plasma Glucose, mg/dL	131 [115, 158]	132 [119, 151]	0.79	138 [122, 157]	129 [113, 145]	<0.01	132 [117, 148]	133 [118, 156]	0.12
Glycated hemoglobin, %	7.1 [6.4, 8.1]	6.8 [6.4, 7.4]	<0.01	7.0 [6.5, 7.6]	6.8 [6.4, 7.3]	0.06	6.8 [6.4, 7.4]	7.0 [6.5, 7.5]	0.23
LDLcholesterol, mg/dL	108 [90, 126]	99 [81, 117]	<0.01	97 [79, 115]	105 [87, 124]	<0.01	101 [81, 118]	101 [83, 118]	0.51
HDL cholesterol, mg/dL	51 [45, 60]	54 [46, 65]	<0.05	51 [44, 61]	58 [49, 67]	<0.01	56 [46, 68]	53 [46, 61]	<0.01
Triglycerides, mg/dL	129 [91, 186]	95 [70, 141]	<0.01	105 [72, 156]	102 [73, 145]	0.61	86 [61, 130]	113 [85, 167]	<0.01
Creatinine, mg/dl	0.78 [0.65, 0.90]	0.86 [0.71, 1.02]	<0.01	0.92 [0.79, 1.09]	0.69 [0.59, 0.82]	<0.01	0.84 [0.69, 1.01]	0.81 [0.68, 0.98]	0.42
eGFR, ml/min/1.73m^2	71.7 ± 19.9	60.0 ± 16.3	<0.01	64.0 ± 18.1	64.7 ± 19.1	0.70	63.1 ± 18.2	65.0 ± 18.8	0.29
**Medications**									
Sulfonylurea, n (%)	15 (9)	28 (10)	0.76	28 (12)	15 (8)	0.19	14 (8)	29 (11)	0.34
Insulin, n (%)	46 (28)	79 (29)	0.96	73 (30)	52 (27)	0.47	56 (33)	69 (26)	0.14
Biguanide, n (%)	101 (62)	123 (45)	<0.01	119 (49)	105 (54)	0.27	72 (42)	152 (57)	<0.01
Glinide, n (%)	30 (19)	83 (30)	<0.01	74 (30)	39 (20)	<0.05	57 (33)	56 (21)	<0.01
Pioglitazone, n (%)	61 (38)	77 (28)	<0.05	76 (31)	62 (32)	0.86	24 (14)	114 (43)	<0.01
α-GI, n (%)	26 (16)	61 (22)	0.13	61 (25)	26 (13)	<0.01	45 (26)	42 (16)	<0.01
DPP4 inhibitor, n (%)	92 (57)	179 (65)	0.94	153 (63)	118 (61)	0.69	106 (62)	165 (62)	0.93
GLP1, n (%)	21 (13)	12 (4)	<0.01	26 (11)	7 (4)	<0.01	9 (5)	24 (9)	0.15
SGLT2 inhibitor, n (%)	47 (29)	48 (17)	<0.01	58 (24)	37 (19)	0.24	26 (15)	69 (26)	<0.01

Median [25%, 75%], Mean ± SD or number (%)
