# Supplementary figures and images for: Walking Speed is the Sole Determinant Criterion of Sarcopenia of Mild Cognitive Impairment in Japanese Elderly Patients with Type 2 Diabetes Mellitus
Source: J Clin Med. 2020 Jul 6;9(7):2133. doi: 10.3390/jcm9072133 (PMC7408848; doi:10.3390/jcm9072133)

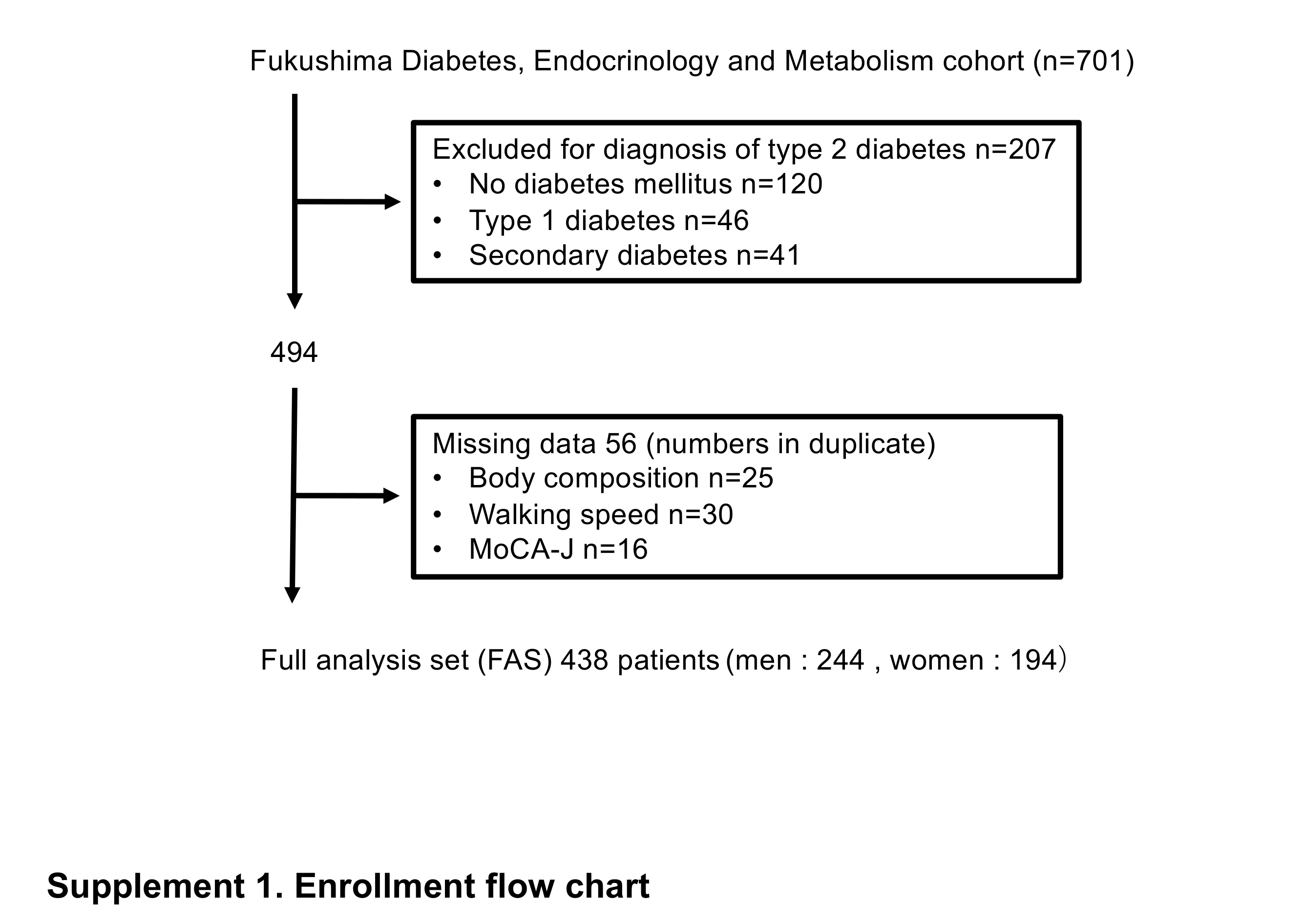

Supplement: Supplementary file 1 [file jcm-09-02133-s001.zip › S1.tif]
